# Characterization of Anti-Bacterial Effect of the Two New Phages against Uropathogenic *Escherichia coli*

**DOI:** 10.3390/v13071348

**Published:** 2021-07-12

**Authors:** Lívia Slobodníková, Barbora Markusková, Michal Kajsík, Michal Andrezál, Marek Straka, Adriána Liptáková, Hana Drahovská

**Affiliations:** 1 Medical Faculty, Institute of Microbiolog, Comenius University in Bratislava, 81108 Bratislava, Slovakia; livia.slobodnikova@fmed.uniba.sk (L.S.); marek.straka@fmed.uniba.sk (M.S.); 2 Department of Molecular Biology, Faculty of Natural Sciences, Comenius University in Bratislava, Ilkovičova 6, 84104 Bratislava, Slovakia; szalaiova.b@gmail.com (B.M.); kajsik.m@gmail.com (M.K.); michalandrezal@gmail.com (M.A.); hana.drahovska@uniba.sk (H.D.); 3 Comenius University Science Park, Ilkovičova 8, 84104 Bratislava, Slovakia

**Keywords:** urinary tract infections, phage therapy, uropathogenic *Escherichia coli*

## Abstract

Urinary tract infections (UTIs) are among the events that most frequently need medical intervention. Uropathogenic *Escherichia coli* are frequently their causative agents and the infections are sometimes complicated by the presence of polyresistant nosocomial strains. Phage therapy is a tool that has good prospects for the treatment of these infections. In the present study, we isolated and characterized two bacteriophages with broad host specificity against a panel of local uropathogenic *E. coli* strains and combined them into a phage cocktail. According to genome sequencing, these phages were closely related and belonged to the *Tequatrovirus* genus. The newly isolated phages showed very good activity on a panel of local clinical *E. coli* strains from urinary tract infections. In the form of a two-phage cocktail, they were active on *E. coli* strains belonging to phylogroups B2 and D, with relatively lower activity in B1 and no response in phylogroup A. Our study is a preliminary step toward the establishment of a national phage bank containing local, well-characterized phages with therapeutic potential for patients in Slovakia.

## 1. Introduction

Urinary tract infections are among the events that most frequently need medical intervention. They can even threaten the life of the patient, especially if complicated with underlying conditions, such as the presence of urinary catheters, stents, urinary stones, transplantations, carcinoma, or diabetes [1,2,3]. These infections are frequently caused by poly-resistant nosocomial strains, which can further complicate the treatment. Urinary pathogens resistant to antibiotics have also been present in the community for decades [4]. Phage therapy, introduced into therapeutic practice in human medicine by D’Herelle at the end of the First World War but neglected with the start of the antibiotic era [5], is a tool that can be used to treat infections caused by poly-resistant bacterial strains. Lytic bacteriophages attack bacteria in a strain-specific manner, independently of their antibiotic resistance profile. Multiplication of therapeutic phages at the site of infection increases the efficiency of bacterial clearance, and all of this happens without a negative effect on the microbiota equilibrium and without the other usual side-effects of antibiotic treatment [6,7]. Phage therapy may also be useful for patients with chronic or recurrent urinary tract infections, which are most common in females. These repeated infections arise after reinfection by urinary pathogens that colonize the intestinal and/or vaginal mucosa or are reactivated from a persisting, silent infectious biofilm focuse in the urinary tract or from the quiescent intracellular reservoirs of uropathogenic bacteria in the deeper layers of the transitional epithelium of the urinary bladder [1,2]. Even though these infections are frequently caused by susceptible bacterial agents, phage therapy may spare the patients repeated courses of antimicrobial therapy and their inevitable side effects. During therapy, bacteriophages can be used as the only therapeutic tool or can be combined with antibiotics (mostly applied systemically, while the phages are applied locally, directly to the infectious focus) [8]. Local intestinal and intravesical application of therapeutic phage preparations can have an immediate antibacterial effect and it can also, with high probability, supplement the microbiota on intestinal and urogenital mucosal surfaces through the use of specific phages, resulting in a prolonged protective effect [9,10]. Phage therapy arises from a long continuous tradition, emerging from the work of the originator of phage therapy, Felix D’Herelle, and later represented by the Eliava Institute of Bacteriophages, Microbiology and Virology in Tbilisi, Georgia. In this country, phage therapy is nowadays a component of standard and routine medical practice for both prophylactic and treatment purposes. Several commercial therapeutic phage cocktails for a variety of applications are freely available for use with a doctor’s advice or for self-treatment of less serious problems. A broader range of products, including personalized bacterial strain-specific therapeutic phage preparations, are available for patients treated in the medical centres [11]. In addition, commercial phage cocktails are also provided by the company NPO Microgen, which has production facilities in the Russian Federation.

As for urinary tract infections, the most common agents, isolated from the urine of infected patients, are uropathogenic strains of *E. coli* [1,3]. Phages active against *E. coli* are included in several commercial phage-cocktail preparations for the treatment of intestinal or pyogenic infections (http://phage.ge/products/, accessed on 17 March 2021; https://www.microgen.ru/en/products/bakteriofagi/, accessed on 17 March 2021). However, they are mostly only available in the countries of their origin (i.e., in Georgia and the Russian Federation). Therefore, the aim of the present study was, in accordance with the trends in phage therapy progress in the EU, to isolate and characterize phages with therapeutic potential that are active against local strains of uropathogenic *E. coli* strains.

## 2. Materials and Methods

### 2.1. Isolation and Molecular Characterization of New Bacteriophages against Uropathogenic Escherichia coli Strains

The phages were isolated from samples from the Bratislava-Petržalka wastewater treatment plant on indicators *E. coli* KMB-507 (genogroup B2, ST420, CH type 38-5) for vB_EcoM_KMB22 phage and *E. coli* KMB-517 (genogroup B2, ST131, CH type 40-41) for vB_EcoM_KMB26 phage. Ten millilitres wastewater, sterilized by passing it through a 22 µm filter, was mixed with the same volume of twofold concentrated LB medium and 200 µL of overnight bacterial culture. The inoculated mixture was cultivated overnight at 37 °C by shaking. Single-species phages were obtained through three repeated isolations from single plaques on double agar followed by ultracentrifugation in CsCl gradient. The isolated phages were preserved long-term in SM buffer (100 mM NaCl; 8 mM MgSO_4_; 50 mM Tris-HCl, pH 7.5; 0.002% gelatine) at 2 to 8 °C for no more than 18 months.

### 2.2. Isolation of Phage DNA, Sequencing and Bioinformatics

Phage DNA was purified using a Phage DNA Isolation Kit (Norgen Biotek, Thorold, ON, Canada). A DNA fragment library was prepared using a Nextera kit (Illumina, San Diego, CA, USA). Paired-end sequencing with 2 × 150 bp reads was carried out on a MiSeq system (Illumina, San Diego, CA, USA). De novo assembly into contigs was carried out on all reads using SPAdes [12] (Center for Algorithmic Biotechnology, St. Petersburg, Russia). The genome was annotated using RAST (http://rast.nmpdr.org/, accessed on 23 April 2020) [13] and Patric server (https://www.patricbrc.org, accessed on 23 April 2020) [14]. The closest relatives of sequenced contigs were found using BLASTn to search the GenBank database and sequences were analyzed manually using Geneious version 11.1.5 (Biomatters Ltd., Auckland, New Zealand). The presence of resistance-encoding genes or virulence genes in the genomes of newly isolated phages was detected using VirulenceFinder tool [15].

### 2.3. Clinical Escherichia coli Strains

Clinical *E. coli* strains were isolated from the urine of hospitalised patients with urinary tract infection. Urine culture, the interpretation of culture results and identification of *E. coli* isolates were performed with routine standardised laboratory methods [16]. Antimicrobial susceptibility to ampicillin, ampicillin/sulbactam, cefuroxime, cefotaxime, ceftazidime, meropenem, gentamicin, ciprofloxacin and co-trimoxazole along with the production of ESBL were tested according to the EUCAST recommendations (https://eucast.org/, accessed on 10 June 2019). Strains were classified into phylogenetic groups by quadruplex PCR [17] and into CH types by two-locus Sanger sequencing [18].

The *E. coli* isolates were preserved in aliquots of Skim-Milk Medium (OXOID, Basingstoke, Hampshire, UK) frozen at −20 °C and freshly revitalized before testing by inoculation and overnight cultivation on blood agar at 35 °C.

### 2.4. Detection of Phage Antibacterial Activity

For determination of the efficiency of plating (EOP), 200 μl of overnight bacterial culture was mixed with 5.0 mL of top agar and overlaid onto the surface of an LB agar plate. Ten microliters of the diluted phage suspension (10^4^–10^8^ PFU/mL) was spotted onto the plate and incubated overnight at 37 °C to obtain visible zones of lysis.

Determination of the phage cocktail activity: bacterial cultures for inoculum preparation were obtained by overnight cultivation of freshly revitalized clinical *E. coli* strains on LB agar. Afterwards, suspensions were prepared in 2 mL aliquots of sterile physiologic solution and were adjusted using a DEN-1 McFarland Densitometer (BioSan, Riga, Latvia) to reach a density of a 0.5 McFarland turbidity standard. The standardised bacterial suspension was poured over the surface of LB agar medium, the excess of suspension was removed and the bacterial inoculum was left to soak into the agar medium for approximately 20 min. The phage cocktail was prepared by mixing vKMB22 and vKMB26 phages to a final concentration of 10^8^ PFU/mL of each phage. The phage suspensions were point-applied in 10 µl volumes on the surface of LB agar medium seeded by the particular tested bacterial strain.

Plaque formation was assessed after overnight cultivation at 35 °C. Antibacterial activity was marked by the presence of confluent bacterial lysis, the presence of semi-confluent plaques, or by the formation of individual isolated plaques in the area of the inoculated spot. Any type of reaction was considered to be positive and the reactions in both dilutions of phage suspensions were taken in account.

## 3. Results

### 3.1. Newly Isolated Anti-Escherichia coli Phages

**Figure 1:**
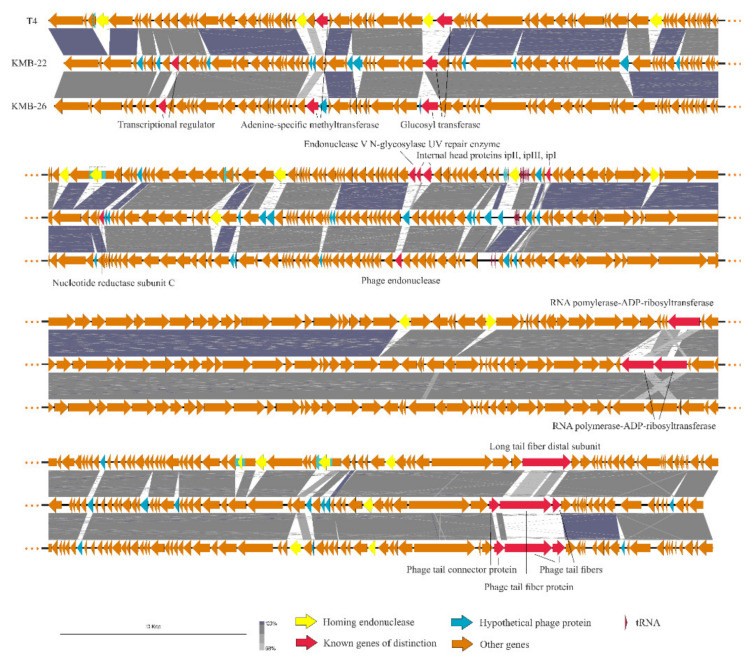
Comparison of phages used in the cocktail with the T4 phage;; analysis and visualisation were conducted in easyfig [23].

Two broad host-spectrum phages were isolated from local wastewater and mixed to compose a phage cocktail for testing with currently circulating *E. coli* strains isolated from urinary tract infections of hospitalised patients. Both phages, vB_EcoM_KMB22 (vKMB22) and vB_EcoM_KMB26 (vKMB26), were classified as members of the Tevenvirinae subfamily of Myoviridae and belonged to the *Tequatrovirus* genus [19]. Up to 88% of their genomes showed homology with the T4 genome (NC_000866.4) [20]; the DNA similarity reached 98% and 95% for vKMB22 and vKMB26, respectively (Figure 1). The main differences between phage genomes were observed in regions encoding for the long tail fibres which are responsible for the host specificity. The vKMB22 tail fibres possessed a similar genetic organization to T4, but the sequence of the distal tail fibre protein greatly differed from T4 homologous gp37, the adhesin responsible for the binding to the host cell surface. The vKMB26 phage showed a higher difference, as it had a unique sequence of three genes corresponding to gp36, gp37 and gp38 of the T4 phage. Whereas, in the T4 phage, the C-terminal domain of gp37 is responsible for interaction with the host receptor, the vKMB26 has the adhesin encoded on a separate protein, gp38, localized downstream of the gp37 gene. Such organization is frequently observed in the Tevenvirinae subfamily (e.g., in the T2 and T6 phages) [21]. Other than tail fibres, further genes with various functions indicated dissimilarities between vKMB22, vKMB26 and the reference T4 phage genome covering transcriptional regulators, ADP-ribosyltransferase, internal head proteins, adenine-specific methyltransferase, glycosyl transferase, nucleotide reductase, phage endonuclease and several proteins with unknown functions. Homing endonucleases were another important source of variability between the phages. Both the vKMB22 and vKMB26 phages contained two endonuclease genes, which are less than 15 present in the T4 phage [22].

### 3.2. Characteristics of Clinical Escherichia coli Strains Selected for Phage-Susceptibility Testing

Forty clinical *E. coli* strains isolated from urinary tract infections were selected for the study (Table 1, Table S1). Fifteen strains were polyresistant ESBL-producers, ten strains were ESBL-negative but resistant to at least one of the tested antibacterial groups and fifteen strains were susceptible to all tested drugs. According to the molecular typing, the stains belonged to four phylogroups: B2 was the most prevalent with 24 strains, followed by groups D (7 strains), A (6 strains) and B1 (3 strains). Nineteen CH types were distinguished; the most prevalent types were 40-41 (six strains), 40-30 (five strains) and 11-54 (five strains). More than half of the strains belonged to internationally spread UPEC clones, e.g., ST131 (CH types 40-30, 40-41), ST73 (CH types 24-10, 24-30, 24-103), ST95 (CH types 38-30) and ST69 (CH types 35-27, 35-47, 35-483) [24].

Fifteen ESBL-producers were included in six different CH types, and nine of them belonged to ST131. Thirteen strains, which were resistant to at least one of the tested antibiotic groups but without ESBL production, belonged to six CH types; four strains were classified into the same phylogroup A and CH type 11-54, but they possessed different resistotypes. With regard to clonality, the fifteen *E. coli* strains susceptible to all tested drugs were the most heterogeneous—they were of twelve different CH types. The ST73 clone formed half of these strains (Table 1, Table S1).

### 3.3. Antibacterial Activity of the Phages

The newly isolated phages were found to possess broad host specificity from in vitro testing. The vKMB22 phage infected 18 of 40 tested strains and 9 of them showed highly efficient infection (EOP > 10%). The vKMB26 phage was even more efficient: it infected 33 of 40 tested strains and 22 of them were efficient hosts (Table 1).

The cocktail composed of vKMB22 and vKMB26 mostly showed similar lysis activity as vKMB26 alone, but with a more pronounced effect against several strains and CH types. The undiluted cocktail was able to lyse 33 strains (82%). Using the hundredfold diluted cocktail, we observed spot lysis in 23 strains (57%). The detailed overview of the phage host specificity is shown in Table S1.

In particular, the cocktail was highly effective against strains belonging to the B2 phylogroup and especially those belonging to the ST131 clone. On the other hand, all strains belonging to the A phylogroup were fully resistant to the new phages. However, only one of these strains was an ESBL producer. The phage cocktail was species-specific and was not able to lyse strains of *Enterobacter* and *Klebsiella* genera (data not shown).

## 4. Discussion

Phage preparation for the treatment of urinary tract infections caused by *E. coli* should utilize phages active against a broad spectrum of uropathogenic *E. coli* strains currently circulating in the geographic area of concern. In the present study, we isolated and characterized phages with therapeutic potential by using a panel of local uropathogenic *E. coli* strains.

Two bacteriophages, isolated from a wastewater treatment plant by using clinical *E. coli* strains as indicators, showed a broad host specificity and were selected for further study. Using genome sequencing, these phages were classified as closely related and belonging to the *Tequatrovirus* genus. T4-related bacteriophages are common in a variety of environments, easy to isolate and belong to the most studied phage groups [19,21,22]. Due to their strictly lytic life they are suitable for phage therapy [25,26], and previous studies have indicated their safety for therapy [27]. No genes encoding for resistance or virulence factors were found in genomes of our phages [15]. Like other *Tequatrovirus* phages, vKMB22 and vKMB26 possessed a mosaic level of relatedness to the reference T4 phage [22]. The tail fiber adhesins are the primary determinants of the host range in *Tequatrovirus* bacteriophages and the sequences of the long tail fiber genes are among the most variable, which was also observed in our study, as the highest diversity relating to vKMB22 and vKMB26 was detected in the long tail fiber distal subunit and tail fiber adhesin genes (Figure 1) [21]. Some other genes encoding for transcriptional regulators, ADP-ribosyltransferase, internal head proteins, adenine-specific methyltransferase and glycosyltransferase showed sequence differences between the vKMB22, vKMB26 and T4 phages. These proteins directly interact with the host targets during the phage life cycle or have a role in inactivating anti-phage host defence, and therefore their variability in closely related phages is not surprising and could also contribute to the host range spectrum. In agreement with these variabilities, the phages had different host specificities: vKMB22 lysed 18 (44%) and vKMB26 33 (82.5%) out of 40 local *E. coli* strains, but their specificity partially overlapped (Table 1 and Table S1). We suppose that the broader host specificity of the vKMB26 phage was partially due to its isolation on the *E. coli* ST131 strain, which belongs to the predominant *E. coli* lineage among extraintestinal pathogenic *E. coli* isolates worldwide [28,29]. The vKMB22 phage was isolated on *E. coli* ST420, which is a rather rare ST of the same B2 phylogroup [28].

The cocktail of two newly isolated phages was tested on the collection of 40 clinical *E. coli* strains. The collection was not too large, but it sufficiently reflected the genetic variability of local *E. coli* from urinary tract infections—it contained 19 different CH types and 12 resistotypes and also covered the internationally spread UPEC clones, including ST131 [24,28,30].

The cocktail showed very good activity on the tested *E. coli* strains. This activity reached 78%, which is in accordance with publicly available data that suggests that the primary resistance of bacteria to commercial phage preparations can be close to 20% [11]. However, lysis with a diluted cocktail was only observed for 23 strains (57%). This value could be a more realistic estimation of the cocktail efficiency in vivo, as it was shown previously that single spot tests overestimate the phage host range [31]. The cocktail showed good activity against strains belonging to phylogroups B2 and D, relatively lower activity in B1 strains and no response in phylogroup A. The strains belonging to this phylogroup are relatively infrequent in UTI and are generally well-susceptible to antibiotic therapy. We supose that bacteriophages active against the phage-resistant strains can be isolated in the future and added into our cocktail.

The bacteriophages from commercial preparations must be regularly tested against a wide range of currently circulating bacterial strains and, if necessary, upgraded by adding new phages against those strains [11,32]. This statement also supports the idea concerning the necessity of relying on phages from local sources, active against local strains of pathogenic bacteria, as was also seen in our study.

We assume that the newly isolated and characterized broad-spectrum anti-*E. coli* phages have therapeutic potential, especially for the treatment of patients coming from the same geographic area. However, factors other than the phage safety and in vitro antibacterial efficacy may interfere with the phage therapy outcome in patients with urinary tract infections. The concentration of bacteriophages at the site of infection (phages may be diluted with urine and disseminated in the bladder), physical access to the infectious focus and bacterial load are important parameters for using and evaluating bacteriophage therapy. Last but not at least, proper patient selection is also a key factor, as suggested in the recently published study by Leitner et al. [33]. The urine itself, being a milieu for *in vivo* bacterial growth in the urinary tract, might also interfere with the final phage effectiveness. The growth of *E. coli* in urine leads to different gene expression compared to the use of LB medium [34], and it cannot be ruled out that the phage receptors may be affected as well. Furthermore, urine may contain natural human antibacterial molecules and immune cells [35] which could support or modify the in vivo activity of phages. Similarly, antimicrobial therapy combined with phage therapy may provide additional benefits [36]. Due to the complexity of these factors, a separate preclinical follow-up study should be performed to clear up these points.

## 5. Conclusions

Two new lytic phages with activity on *Escherichia coli* were isolated, identified and characterized. Based on the in vitro studies, the phages have therapeutic potential for the treatment of urinary tract infections caused by *E. coli* strains with various antimicrobial resistance profiles and clonality. After the implementation of the legal aspects of phage therapy in the framework of healthcare legislation in Slovakia, this new phage cocktail may potentially fill a gap in the therapeutic armamentarium for the treatment of urinary tract infections in patients infected by polyresistant strains of *E. coli*, allergic to antibacterial drugs or suffering from repeated or persistent urinary tract infections. Afterwards, as part of experimental therapy, the in vivo therapeutic potential of newly isolated phages could be confirmed as well.

Through the isolation of new phages against *E. coli* strains, a collection of phages with potential uses in human medicine has been established, which will be successively enriched by phages active against clinical *E. coli* strains that are resistant to available phage cocktails, as well as against the other medically important bacterial species. This will result in a national phage bank establishment that will serve as a source of phages for therapeutic use in the form of cocktails with fixed formulations or a personalised phage therapy.

## Figures and Tables

**Table 1:** Sensitivity of *Escherichia coli* strains to the new phages and their cocktail.

				No. of Sensitive Strains ^2^		
Genomic Group	CH Type ^1^	No. of Strains	No. of AMR Strains (ESBL)	vKMB22	vKMB26	Cocktail
A	11–54	5	5 (1)	0/0	0/0	0/0
	11–23	1	0 (0)	0/0	0/0	0/0
B1	6–31	2	1 (0)	0/0	1/2	1/2
	4–54	1	0 (0)	0/0	0/1	0/1
B2	13–5	1	0 (0)	0/0	1/1	1/1
	13–233	1	1 (0)	0/0	1/1	1/1
	14–27	1	0 (0)	0/0	1/1	1/1
	24–10	3	2 (2)	1/1	1/3	3/3
	24–103	1	0 (0)	0/1	1/1	1/1
	24–30	4	0 (0)	0/0	4/4	4/4
	38–30	1	1 (1)	0/1	1/1	1/1
	40–30	5	5 (4)	2/5	1/5	4/5
	40–41	6	5 (5)	3/6	5/6	4/6
	52–5	1	0 (0)	0/0	0/1	0/1
D	26–0	2	2 (0)	2/2	2/2	2/2
	35–27	1	1 (0)	0/0	0/1	0/1
	35–47	1	0 (0)	0/1	1/1	0/1
	35–483	1	0 (0)	0/0	0/0	0/0
	37–56	2	2 (1)	1/1	2/2	0/2

^1^ Strains belonged to the main UPEC clones ST73 (24–10, 24–30, 24–103), ST95 (38–30), ST131 (40–30, 40–41) and ST69 (35–27, 35–47, 35–483) [24]; AMR strains—antimicrobial resistant strains; ESBL—producers of extended spectrum beta-lactamases; ^2^ numbers of highly sensitive strains/all sensitive strains are shown.

## Data Availability

The data for this study have been deposited in the European Nucleotide Archive (ENA) at EMBL-EBI under accession number PRJEB44945 (https://www.ebi.ac.uk/ena/browser/view/PRJEB44945; accessed on 12 July 2021) or in the shared folder https://www.dropbox.com/s/oq3zwzaj2hqik2z/Seq.zip?dl=0 (accessed on 12 July 2021).

## Supplementary Materials

The following are available online at https://www.mdpi.com/article/10.3390/v13071348/s1, Table S1: Characteristics of the clinical *Escherichia coli* strains panel.
